# Leadership and capacity building in chiropractic research: report from the first CARL cohort

**DOI:** 10.1186/s12998-021-00363-8

**Published:** 2021-02-22

**Authors:** Jan Hartvigsen, Greg Kawchuk, Alexander Breen, Diana De Carvalho, Andreas Eklund, Matthew Fernandez, Martha Funabashi, Michelle M. Holmes, Melker S. Johansson, Katie de Luca, Craig Moore, Isabelle Pagé, Katherine A. Pohlman, Michael S. Swain, Arnold Y. L. Wong, Jon Adams

**Affiliations:** 1 Department of Sports Science and Clinical Biomechanics, University of Southern Denmark, Odense, Denmark; 2 Nordic Institute of Chiropractic and Clinical Biomechanics, University of Southern Denmark, Odense, Denmark; 3 Faculty of Rehabilitation Medicine, University of Alberts, Edmonton, Canada; 4 AECC University College, Bournemouth, UK; 5 Faculty of Medicine, Memorial University of Newfoundland, St. John’s, NL Canada; 6 Institute of Environmental Medicine, Karolinska Institutet, Stockholm, Sweden; 7 Department of Chiropractic, Macquarie University, Sydney, Australia; 8 Division of Research and Innovation, Canadian Memorial Chiropractic College, Toronto, Canada; 9 Department of Chiropractic, Université du Québec à Trois-Rivières, Trois-Rivières, Québec Canada; 10 School of Psychology, University of Southampton, Southampton, UK; 11 Research Institute, Parker University, Dallas, TX USA; 12 Department of Rehabilitation Sciences, The Hong Kong Polytechnic University, Hong Kong, Hong Kong; 13 School of Public Health, University of Technology Sydney, Sydney, Australia

**Keywords:** Research capacity, Leadership, Chiropractic

## Abstract

The Chiropractic Academy for Research Leadership (CARL) was formed in 2016 in response to a need for a global network of early career researchers and leaders in the chiropractic profession. Thirteen fellows were accepted competitively and have since worked together at residentials and virtually on many research and leadership projects. In 2020, the CARL program ended for this first cohort, and it is now timely to take stock and reflect on the achievements and benefits of the program. In this paper we present the structure of CARL, the scientific and leadership outputs as well as the personal value of CARL for the participating fellows. As a result of the success of the first CARL cohort, organizations from Europe, North America, and Australia have supported a second cohort of 14 CARL fellows, who were competitively accepted into the program in early 2020.

**Supplementary Information:**

The online version contains supplementary material available at 10.1186/s12998-021-00363-8.

## Background

The Chiropractic Academy for Research Leadership (CARL) was conceived in late 2015 by three senior academics (Adams, Kawchuk, Hartvigsen) to identify and mentor talented, promising early career chiropractic researchers in a global network [1]. The program was devised in response to a lack of mature research culture across the chiropractic profession and aimed to develop a critical mass of successful early-career chiropractic researchers globally while at the same time encourage multi-disciplinary perspectives and cooperation in research and academia across professions. In parallel, CARL was additionally intended to be an opportunity for a ‘timeout’ from the day-to-day pressure of work environments allowing space to reflect and consider long-term, deeper issues around individual career development, as well as strategic research planning for the chiropractic profession. Importantly, the founders aimed to develop confidence amongst these future leaders by providing direct mentorship, support and advice regarding research focus, personal and skills development, and career pathways.

In late 2015 an international call for applications was launched. Through a competitive process that involved assessments of written applications (including motivations, experience and expertise) and personal interviews, 13 CARL fellows were accepted into the program. Three years later in June 2020, CARL ended for this first cohort and a second cohort of Fellows, CARL II, was accepted using the same procedure.

The overall aim of this paper is to report on the process and outcomes of the first cohort of CARL fellows, those participating in the program between 2016 and 2020. Specifically, we aim to describe the content and activities of the three-year program, its scientific and leadership outputs, the contextual and informal impact of the program for the fellows and mentors, and finally the implications for the chiropractic profession.

## Main text

Here we provide an overview of the structure and outputs from the first cohort in the Chiropractic Academy for Research Leadership (CARL) program. A detailed list of outputs can be found in Additional file 1.

### Structure of CARL – residentials and online meetings

The CARL program is structured around annual week-long residentials rotating each year between Denmark, Canada, Australia (mentor’s universities) and in connection with relevant international conferences or meetings. The inaugural CARL residential was held in April of 2017 at the campus of the University of Southern Denmark, Odense, Denmark (Hartvigsen); the second was held in April of 2018 at the campus of the University of Alberta, Edmonton, Canada (Kawchuk); the third was held in March of 2019 in Berlin, Germany, prior to the 2018 World Federation of Chiropractic bi-annual conference; and the fourth residential was to be held in 2020 on the campus of the University of Technology Sydney, Sydney, Australia (Adams) but was postponed due to the COVID-19 pandemic. In between residentials, there were regularly scheduled videoconferences for all fellows and mentors as well as numerous teleconferences of sub-groups based on specific research and leadership activities.

The residentials were structured into blocks of guest speakers, group discussions, worktime and common physical activities. Guest speakers have included a range of successful early career and senior academics from different fields who have presented and discussed diverse topics with the fellows such as career planning, work-life balance in academia, women in research, emerging technologies, and research about management and leadership. Some guest speakers have also been from industry including the CEO of a Canadian provincial bank. Group discussions have spanned similar issues but focused at a personal level and in confidence. One recurrent session throughout all the residentials has been the “*Problem Solved”* session where each fellow presents a challenge or problem to the group, and the group then reflects and discusses potential solutions. Challenges can be work-related or from personal life. Another recurrent session has been *collective brainstorming* sessions about research and leadership projects where fellows and mentors present and discuss ideas and possibilities often based on existing datasets or upcoming collaborative opportunities. The residentials have also contained substantial periods for working together in groups to develop and advance projects design, data analyses, and manuscript writing. Finally, one session every day has included physical activity ranging from indoor football, Zumba (a recurring favorite) and organized walks through unique areas of the hosting city.

## Results and outputs from CARL

A CARL publication was defined as a paper published as a result of a collaborative CARL project with at least two fellows and a mentor as co-authors. CARL projects collectively produced 38 international peer-reviewed papers published in 20 different journals with an average impact factor (IF) of 2.582 (if the journal had an IF). A CARL conference presentation was similarly defined, with CARL fellows having a total of 81 conference abstracts accepted (43 podium presentations, 32 posters and 6 workshops). Of the 24 conferences where CARL fellows presented, 10 were national and 14 were international meetings (Table 1).

**Table 1:** CARL research production as documented by publications, conference presentations and research funding received between April 25, 2017 and June 1, 2020

International Peer-Reviewed Publications	
Total publications	38
# of unique journals	20
# of journals with impact factor	12
Average impact factor (*n* = 12)	2.582
Weighted impact factor (*n* = 18)	2.330
Conference Presentations	
Total accepted conference abstracts	84
Total unique conferences	24
# of local/national conferences	10
# of international conferences	14
# of podium presentations	43
# of poster presentations	32
# of workshop presentations	6
Submitted abstracts with no acceptance result due to COVID-19 conference cancellations	3
Research Funding	
Travel grants and scholarships (USD)	$ 39,954.72
Research grants (USD)	$ 519,411.57

A broad range of leadership activities originated from CARL including 15 conference organizations, 18 memberships of councils and/or boards, 10 editorial board memberships, three themed meetings about women in chiropractic, conveners in research sessions at conferences, media activity about CARL and academic course or workshop leadership (Table 2).

**Table 2:** CARL leadership activities between April 25, 2017 and June 1, 2020

	Frequency		Fellows Involved	
Conference Organization Committees		15		10
Council and Board memberships		18		9
Editorial Boards		10		5
Women in Chiropractic Research		3		7
Research Conveners		3		4
Media promotion of CARL		4		3
Led a course/workshop		2		2

The 13 CARL fellows were awarded a total of US $559,366.29 in research or travel grants during the 3 years (2016–2020). Six CARL fellows were awarded PhD degrees while participating on the program. Collectively, the fellows received 17 research awards, and 14 new professional academic appointments or promotions were obtained amongst the fellows since their participation on the program (Table 3). For a detailed list of CARL outputs, see Additional file 1.

**Table 3:** Individual CARL fellow professional accomplishments between April 25, 2017 and June 1, 2020

Professional Accomplishments	
PhD degrees earned	6
Professional positions (new and promotions)	14
Research Awards	17

### Personal value of CARL

At the end of this three-year program, all fellows were given an opportunity to reflect on the value of the CARL program in writing. Feedback was analyzed and collated by one of the fellows with expertise in qualitative methodology (Holmes). Several themes emerged such as CARL fellows feeling that the program has had a substantial impact on their life: “*it has made me a better person and a better researcher*.” CARL fellows discussed gaining confidence as early career researchers (ECRs) and overall personal growth: *“CARL has given the individual fellows leadership and mentorship skills to develop into mature academics.”.* CARL fellows acknowledged the professional development and opportunities that CARL had opened up for them, advancing their career: *“The program has been a catalyst and allowed me to make major leaps in my development in multiple areas, leaps that otherwise wouldn’t have been possible or taken much longer to achieve.”.* Overall, CARL was seen to be valuable for the chiropractic profession: *“CARL has created young leaders for the profession, aspirational role models for the next generation of chiropractic researchers.”.*

CARL fellows also appreciated the supportive nature of CARL: “*The program provided support and direction at a vulnerable career stage*.”. Many fellows highlighted the difficulties they faced as ECRs prior to CARL, including feeling isolated and frustrated in academia, having a lack of confidence, and doubting one’s self and abilities as a researcher. However, CARL provided a platform for advice, support, celebrating successes, comfort during failures, decision making, and solving challenges: “*CARL is the glue that keeps my professional life together*”. The support received from the mentors, exceeded fellows’ initial expectations: “*CARL has given me the opportunity to learn from research mentors I could only dream of working with*”. The fellows reflected that mentors provided research collaborations, individual support, and advice from their own personal academic careers.

In addition, the CARL fellows valued the personal relationships that had developed between them while participating on the program – providing a “*life-long network of peer mentors*”, described as one of the most valued benefits of the program. As well as providing advice and support, such network also allowed fellows to engage in new methodologies, broaden their understanding of the chiropractic profession, and to develop new, international research project collaborations. Of equal importance, the fellows also valued the friendship that had emerged from the program: *“CARL gave me true friends, who support me not only professionally, but also in my personal life*”.

## Discussion

Upon the completion of the first CARL cohort, we have presented its structure, output and results. The selection of an international group of ambitious and talented ECRs that are mentored together in an innovative, supportive environment, has resulted in significant research and leadership output as well as a coherent and tightly knit network of successful investigators who are realizing their potential as established academics.

It cannot be over emphasized that the choice of an academic career places ECRs at risk of vulnerability through isolation and reclusion. Workdays for most involve substantial teaching loads, academic administration, reporting, and corresponding with limited time and flexibility to read, write, collect and synthesize data, attend meetings and conferences, and connect with fellow academics from diverse backgrounds. Consequently, academics, and especially those in the early stages of their careers face substantial challenges with respect to job progression, performance pressure, high expectations and imbalances between work and life outside of work. Added to these challenges are additional issues for those CARL Fellows who are women, minorities and/or who lack chiropractic peers at their home academic institutions. All of these factors combine to create tremendous pressure on ECRs who must be highly successful to compete for a decreasing number of available academic positions in competition with other highly qualified candidates [2] all while balancing career expectations with family life and other demands upon their time.

While the CARL program cannot solve all issues faced by chiropractic ECRs (no one initiative can aim to do so), it does nevertheless facilitate opportunities for participants to share challenges balancing research and teaching obligations and to establish their own global network of support, which is typically unavailable in just one institution. Such a network helps fellows increase their research productivity and strengthen their chances of advancing to a mid-career, senior and leadership position/role.

Although building a network of peers may be possible within a group of motivated ECRs, mentorship is a vital component of the success of CARL fellows as there are relatively few senior academics in the profession and likely fewer in close proximity to ECRs. An effective mentorship relationship includes both career support and psychosocial support [3], with both of these areas being a focus of the CARL program, especially during the residencies. While mentorship is often thought of as dyadic relationship, the first CARL program represented a successful collective mentorship configuration with the three mentors and 13 mentees, resulting in a triadic structure with bidirectional engagement between fellows and mentors as well as peer-to-peer support between the fellows.

In addition to growing the research capacity of the chiropractic profession, CARL can also be a catalyst that nudges chiropractic towards a mainstream evidence-based model of healthcare that is integrative and collaborative across medical and allied-health settings. Brosnan describes these types of academic outputs as social capital, which can impact the construction of professional identity and guide the future direction of the profession [4]. Indeed, the research outputs generated by the first cohort of CARL fellows have helped towards strengthening and consolidating the professional legitimacy of chiropractic [5] while providing a much-needed (altruistic) service to the benefit of the public [6].

Research is the currency needed for the chiropractic profession to claim the cultural authority it seeks to achieve [7]. Accordingly, there is a strong need for a greater number of chiropractic graduates to be inspired to undertake a research path that can strengthen the professions’ academic capability. Recent studies have identified that only a very small percentage of the chiropractic profession have sought to undertake higher degree research training [8]. While the reasons for this remain unclear, we speculate that it could be in part due to the paucity of funding opportunities to pursue a PhD, i.e. scholarships and research stipends, without additional employment, as well as the lack of job prospects both within and outside traditional and competitive academic positions. While balanced academic positions, i.e. teaching and research, are challenging to find for academics generally, they are even more challenging to find for chiropractors because the chiropractic profession worldwide typically functions outside of public universities. Over a few short years the CARL program, through providing expert guidance, support and the experience of senior academic mentors, has enabled a small group of early career researchers to better understand how to juggle these multiple (and demanding) responsibilities while still delivering quality research.

Not only has the first 3 years of the CARL program been successful in terms of tangible output and metrics, the program has been able to raise support for its next cohort, CARL II. Importantly, sufficient financial resources are a prerequisite for CARL as ECRs would not have the individual funding to be part of this network otherwise. In addition to retaining the original partners and sponsors of the first CARL cohort of fellows, other sponsors have now also joined in supporting the program and securing the next 4 years of CARL residentials and initiatives. These resources also indirectly help the CARL I cohort to stay involved with the program and create opportunities for them to directly increase research capacity and thereby expanding the CARL footprint. Although the challenges of COVID-19 have delayed plans to have a transition meeting between the two cohorts, the possibility remains of not only involving CARL I fellows in the mentorship of CARL II fellows, but also the possibility of employing CARL I fellows as an additional resource to mentor other ECRs in their local jurisdictions.

While resources for CARL II are in place for the next 4 years, the recent challenges of COVID-19 have meant in-person annual residentials let alone attend international meetings have not been possible throughout 2020. Presently, adaptation to the COVID-19 challenge has resulted in more regular virtual meetings as well as scheduled virtual drop-in sessions for CARL fellows to remain in more frequent contact. Intermediate residential strategies are also planned which will allow fellows in the same geographic region to connect in person (local travel conditions permitting) prior to being allowed to travel further afield to international meetings.

## Conclusion

The first cohort of the CARL program have met at yearly residentials and more regularly via videoconference. CARL has significantly contributed to the global research capacity in chiropractic through publication of original scientific papers, conference presentations, and leadership activities. Many CARL fellows have completed PhD theses, achieved employment in or promotion to new academic positions, and have attracted significant competitive research funding. Importantly, the CARL fellows have become a tightly knit, supportive group of successful and highly capable early career researchers who have the potential to further grow and shape the global research agenda for chiropractic over future decades.

## Supplementary Information

**Additional file 1.** Detailed list of journals where CARL publications have been published and/or submitted as of July 1, 2020.

## Data Availability

Not applicable.

## Abbreviations

CARL: Chiropractic Academy for Research Leadership
CV: Curriculum Vitae
CEO: Chief Executive Officer
ECR: Early Career Researcher

## References

[1] Adams J, Kawchuk G, Breen A, De Carvalho D, Eklund A, Fernandez M, Funabashi M, Holmes MM, Johansson MS, de Luca K (2018). Leadership and capacity building in international chiropractic research: introducing the chiropractic academy for research leadership (CARL). Chiropr Man Therap.

[2] Hawkins R, Manzi M, Ojeda D (2014). Lives in the making: power, Academia and the Everyday. ACME.

[3] Haggard DL, Dougherty TW, Turban DB, Wilbanks JE (2011). Who is a Mentor? A review of evolving definitions and implications for research. J Manag.

[4] Brosnan C (2017). Alternative futures: fields, boundaries, and divergent professionalisation strategies within the chiropractic profession. Soc Sci Med.

[5] Myburgh C, Hartvigsen J, Grunnet-Nilsson N (2008). Secondary legitimacy: a key mainstream health care inclusion strategy for the Danish chiropractic profession?. J Manip Physiol Ther.

[6] Welie JV (2004). Is dentistry a profession? Part 1. Professionalism defined. J Can Dent Assoc.

[7] Brown R (2013). Climate change: global challenges for the chiropractic profession (Perspective Report). J Can Chiropr Assoc.

[8] Adams J, Peng W, Steel A, Lauche R, Moore C, Amorin-Woods L, Sibbritt D (2017). A cross-sectional examination of the profile of chiropractors recruited to the Australian chiropractic research network (ACORN): a sustainable resource for future chiropractic research. BMJ Open.

