# Correction to: Leadership and capacity building in chiropractic research: report from the first CARL cohort

**DOI:** 10.1186/s12998-021-00369-2

**Published:** 2021-03-25

**Authors:** Jan Hartvigsen, Greg Kawchuk, Alexander Breen, Diana De Carvalho, Andreas Eklund, Matthew Fernandez, Martha Funabashi, Michelle M. Holmes, Melker S. Johansson, Katie de Luca, Craig Moore, Isabelle Pagé, Katherine A. Pohlman, Michael S. Swain, Arnold Y. L. Wong, Jon Adams

**Affiliations:** 1 Department of Sports Science and Clinical Biomechanics, University of Southern Denmark, Campusvej 55, 5230 Odense M, Denmark; 2 Nordic Institute of Chiropractic and Clinical Biomechanics, University of Southern Denmark, Odense, Denmark; 3 Faculty of Rehabilitation Medicine, University of Alberts, Edmonton, Canada; 4 AECC University College, Bournemouth, UK; 5 Faculty of Medicine, Memorial University of Newfoundland, NL, St. John’s, Canada; 6 Institute of Environmental Medicine, Karolinska Institutet, Stockholm, Sweden; 7 Department of Chiropractic, Macquarie University, Sydney, Australia; 8 Division of Research and Innovation, Canadian Memorial Chiropractic College, Toronto, Canada; 9 Department of Chiropractic, Université du Québec à Trois-Rivières, Trois-Rivières, Québec, Canada; 10 School of Psychology, University of Southampton, Southampton, UK; 11 Research Institute, Parker University, Dallas, TX USA; 12 Department of Rehabilitation Sciences, The Hong Kong Polytechnic University, Hong Kong, Hong Kong; 13 School of Public Health, University of Technology Sydney, Sydney, Australia

**Correction to: Chiropr Man Therap 29, 9 (2021)**

**https://doi.org/10.1186/s12998-021-00363-8**

Following publication of the original article [1], we were notified of a mismatch between the submitted author affiliations (3 to 12) and the published ones.

Originally published affiliations:

- 3. Department of Rehabilitation Sciences, The Hong Kong Polytechnic University, Hong Kong, Hong Kong.
- 4. Faculty of Rehabilitation Medicine, University of Alberts, Edmonton, Canada.
- 5. AECC University College, Bournemouth, UK.
- 6. Faculty of Medicine, Memorial University of Newfoundland, St. John’s, NL, Canada.
- 7. Institute of Environmental Medicine, Karolinska Institutet, Stockholm, Sweden.
- 8. Department of Chiropractic, Macquarie University, Sydney, Australia.
- 9. Division of Research and Innovation, Canadian Memorial Chiropractic College, Toronto, Canada.
- 10. Department of Chiropractic, Université du Québec à Trois-Rivières, Trois-Rivières, Québec, Canada.
- 11. School of Psychology, University of Southampton, Southampton, UK.
- 12. Research Institute, Parker University, Dallas, TX, USA.

Corrected affiliations:

- 3. Faculty of Rehabilitation Medicine, University of Alberts, Edmonton, Canada.
- 4. AECC University College, Bournemouth, UK.
- 5. Faculty of Medicine, Memorial University of Newfoundland, St. John’s NL, Canada.
- 6. Institute of Environmental Medicine, Karolinska Institutet, Stockholm, Sweden.
- 7. Department of Chiropractic, Macquarie University, Sydney, Australia.
- 8. Division of Research and Innovation, Canadian Memorial Chiropractic College, Toronto, Canada.
- 9. Department of Chiropractic, Université du Québec à Trois-Rivières, Trois-Rivières, Québec, Canada.
- 10. Schoolof Psychology, University of Southampton, UK.
- 11. Research Institute, Parker University, Dallas, Texas, United States.
- 12. Department of Rehabilitation Sciences, The Hong Kong Polytechnic University, Hong Kong.

The original article has been corrected.
