# Supplementary material for: Leadership and capacity building in chiropractic research: report from the first CARL cohort
Source: Chiropr Man Therap. 2021 Feb 22;29:9. doi: 10.1186/s12998-021-00363-8 (PMC7897891; doi:10.1186/s12998-021-00363-8)
Supplement: Supplementary file 1 — Additional file 1. Detailed list of journals where CARL publications have been published and/or submitted as of July 1, 2020. [file 12998_2021_363_MOESM1_ESM.docx]

**Appendix 1**

Detailed list of journals where CARL publications have been published and/or submitted as of July 1, 2020.

| **Journal** | **Published** | **Submitted** |
| --- | --- | --- |
| The Spine Journal | 1 | 1 |
| Journal of the Canadian Chiropractor’s Association | 1 | 1 |
| Chiropractic & Manual Therapies | 6 | 5 |
| Musculoskeletal Science and Practice |  | 1 |
| Advances in Integrative Medicine | 1 |  |
| Journal of Manipulative and Physiological Therapeutics | 4 |  |
| Complementary Therapies in Medicine | 3 |  |
| Journal of Chiropractic Education | 2 |  |
| JBI Database of Systematic Reviews and Implementation Reports | 1 |  |
| The Journal of Pain | 1 |  |
| European Journal of Pain | 1 |  |
| Australasian Journal on Ageing | 1 |  |
| BMC Neurology | 1 |  |
| European Spine Journal | 1 |  |
| Gait & Posture | 1 |  |
| Australian Journal of Rural Health | 1 |  |
| Chiropractic Journal of Australia | 1 |  |
| Scientific Reports | 1 |  |
| BMC Complementary and Alternative Medicine | 1 |  |
| Spine | 1 |  |

Detailed list of conference presentations presented by CARL fellows as of July 1, 2020. Not included are three abstracts submitted to the 21^st^ Biennial Meeting of the Canadian Society of Biomechanics (Montreal, Canada 2020) which was postponed due to Covid-19.

| **Conference** | **Location, year** | **Podium** | **Poster** | **Workshop** |
| --- | --- | --- | --- | --- |
| 27th annual scientific poster contest at the Université du Québec à Trois-Rivières | Trois-Rivières, Canada, 2020 |  | 1 |  |
| online edition of the Université du Québec à Trois-Rivières chiropractic department scientific event | Trois-Rivières, Canada, 2020 | 1 |  |  |
| European Chiropractors’ Union Convention | Utrecht, The Netherlands, 2020 |  | 1 |  |
| SpineWeek 2020 | Melbourne, Australia, 2020 | 4 | 4 |  |
| 27^th^ Association of Chiropractic Colleges – Research Agenda Conference (ACC-RAC) | San Diego, USA, 2020 | 7 | 1 | 2 |
| 50th Annual Conference of the Association of Canadian Ergonomists | St. John’s, Canada, 2019 | 1 |  |  |
| Chiropractic Australia National Conference | Gold Coast, Australia, 2019 | 1 | 2 |  |
| Australian Chiropractors Association National Conference | Melbourne, Australia, 2019 | 1 | 2 |  |
| 10th Interdisciplinary World Congress on Low Back & Pelvic Girdle Pain | Antwerp, Belgium, 2019 | 2 |  |  |
| XVIth International Forum on Back and Neck Pain Research in Primary Care | Quebec City, Canada, 2019 |  | 4 |  |
| World Federation of Chiropractic 2019 Congress | Berlin, Germany, 2019 | 8 | 4 | 1 |
| Australian Chiropractic Association – scientific symposium | Hobart, Australia, 2018 | 1 |  |  |
| World Federation of Chiropractic-Association of Chiropractic Colleges, 10TH Chiropractic Education Conference | London, UK, 2018 |  |  | 1 |
| 8th World Congress of Biomechanics | Dublin, Republic of Ireland, 2018 | 1 |  |  |
| The International Back and Neck Pain Research Forum | Oslo, Norway, 2017 |  | 2 |  |
| 11th Pan-Pacific Conference on Rehabilitation, | Hong Kong, 2018 |  | 1 |  |
| Chiropractic Australia National Conference | Sydney, Australia, 2018 | 1 | 1 |  |
| Canadian Chiropractic Association’s National Convention and Tradeshow | Calgary, Canada, 2018 | 2 |  |  |
| International Society for the Studies of the Lumbar Spine (ISSLS) Annual Meeting, | Banff, Canada, 2018 |  | 1 |  |
| Integrative Research Congress: Integrative Medicine & Health | Baltimore, USA, 2018 |  | 1 |  |
| CA-COCA National Conference | Melbourne, Australia, 2017 | 4 | 1 |  |
| Chiropractors’ Association of Australia (CAA) National Conference Research Symposium | Canberra, Australia, 2017 | 3 | 1 |  |
| Musculoskeletal, Bone & Joint Health Alliance | Sydney, Australia, 2017 | 1 |  |  |
| World Congress Integrative Medicine & Health | Berlin, Germany, 2017 |  | 2 |  |
